# Refining Prognosis in Chemoembolization for Hepatocellular Carcinoma: Immunonutrition and Liver Function

**DOI:** 10.3390/cancers13163961

**Published:** 2021-08-05

**Authors:** Lukas Müller, Felix Hahn, Aline Mähringer-Kunz, Fabian Stoehr, Simon Johannes Gairing, Friedrich Foerster, Arndt Weinmann, Peter Robert Galle, Jens Mittler, Daniel Pinto dos Santos, Michael Bernhard Pitton, Christoph Düber, Roman Kloeckner

**Affiliations:** 1 Department of Diagnostic and Interventional Radiology, University Medical Center of the Johannes Gutenberg University Mainz, 55131 Mainz, Germany; lukas.mueller@unimedizin-mainz.de (L.M.); felix.hahn@unimedizin-mainz.de (F.H.); aline.maehringer-kunz@unimedizin-mainz.de (A.M.-K.); fabian.stoehr@unimedizin-mainz.de (F.S.); michael.pitton@unimedizin-mainz.de (M.B.P.); christoph.dueber@unimedizin-mainz.de (C.D.); 2 Department of Internal Medicine, University Medical Center of the Johannes Gutenberg University Mainz, 55131 Mainz, Germany; simonjohannes.gairing@unimedizin-mainz.de (S.J.G.); friedrich.foerster@unimedizin-mainz.de (F.F.); arndt.weinmann@unimedizin-mainz.de (A.W.); peter.galle@unimedizin-mainz.de (P.R.G.); 3 Department of General, Visceral and Transplant Surgery, University Medical Center of the Johannes Gutenberg University Mainz, 55131 Mainz, Germany; jens.mittler@unimedizin-mainz.de; 4 Department of Radiology, University Hospital of Cologne, 50937 Cologne, Germany; daniel.pinto-dos-santos@uk-koeln.de

**Keywords:** hepatocellular carcinoma, transarterial chemoembolization, risk prediction, albumin-bilirubin grade, prognostic nutritional index

## Abstract

**Simple Summary:**

The combination of the albumin-bilirubin (ALBI) grading and the Prognostic Nutritional Index (PNI) offers potential as a highly predictive tool for patients with hepatocellular carcinoma (HCC). The present study evaluated this combination firstly for patients undergoing transarterial chemoembolization (TACE). Both the ALBI grade and PNI were strong independent predictors of survival. However, the combination of the two scores allowed for even more precise predictions. In addition, this new ALBI-PNI outperformed several established scoring systems. Thus, the easy-to-calculate ALBI-PNI may be a promising stratification tool for patients with HCC undergoing TACE in daily clinical routine.

**Abstract:**

A combination of albumin-bilirubin (ALBI) grading and the Prognostic Nutritional Index (PNI) was identified recently as a highly predictive tool for patients with hepatocellular carcinoma (HCC) undergoing tumor ablation. The present study evaluated this combination in patients undergoing transarterial chemoembolization (TACE). Between 2010 and 2020, 280 treatment-naïve patients were retrospectively identified. The influence of ALBI grade, PNI and the novel ALBI-PNI on the median overall survival (OS) was assessed. In the next step, the prognostic ability of the combined approach was compared to established scoring systems. Both ALBI grade 2−3 and a low PNI were highly predictive for median OS (ALBI grade 1–3: 39.0 vs. 16.3 vs. 5.4 months, *p* < 0.001; high vs. low PNI: 21.4 vs. 7.5, *p* < 0.001). The combination of both resulted in a median OS of 39.0, 20.1, 10.3, and 5.4 months (*p* < 0.001). With a Concordance Index (C-Index) of 0.69, ALBI-PNI outperformed each individual score (ALBI 0.65, PNI 0.64) and was also better than BCLC, HAP, mHAP-II, and the Six-and-Twelve score (C-Indices 0.66, 0.60, 0.59, and 0.55). Thus, the easy-to-calculate ALBI-PNI may be a promising stratification tool for patients with HCC undergoing TACE, reflecting both immunonutritive status and liver function.

## 1. Introduction

Hepatocellular carcinoma (HCC) is one of the most common, and deadliest, cancers [1,2]. According to the European Association for the Study of the Liver (EASL) and American Association for the Study of Liver Diseases (AASLD) guidelines, the Barcelona Clinic Liver Cancer (BCLC) staging system is the preferred model for prognostication and treatment allocation [1,2]. According to this framework, TACE is the standard of care for patients with intermediate-stage cancer [3]. However, due to considerable differences in tumor burden and liver function, this stage comprises a heterogeneous subgroup [4,5,6,7]. Furthermore, TACE may be a therapeutic option outside the intermediate stage following the concept of stage migration, further increasing the heterogeneity of the subgroup [1,8]. Thus, prognosis prediction is difficult in these patients. Several models for survival prediction and treatment stratification have been developed, but they all failed in external validation attempts [9,10,11,12,13,14,15,16,17,18,19,20,21]. This creates a need for better stratification tools.

Most patients develop HCC due to liver cirrhosis. Therefore, these patients have two diseases, HCC and liver cirrhosis, leading to impaired liver function. Liver function is historically assessed using the Child-Pugh Score (CPS) [22]. This score combines albumin, the International Normalized Ratio (INR), bilirubin, ascites, and hepatic encephalopathy. However, the subjectivity of the latter factors impairs the reproducibility of this score. Therefore, a new system only based on laboratory parameters was introduced with the albumin-bilirubin (ALBI) grade [23]. In addition to a good performance among patients with liver cirrhosis, the ALBI grading was found to be highly predictive in patients with HCC undergoing different treatment approaches [24]. For patients undergoing TACE, the ALBI grade offers an excellent alternative to the CPS [25,26,27,28]. However, stratification based solely on ALBI grading was not found to be superior to other existing scoring systems for predicting median overall survival (OS) in TACE patients [12].

Another aspect of the combined diseases is the chronic inflammation in the damaged liver, which creates a special microenvironment that influences the patient’s whole immune system and nutrition [29,30]. Thus, assessing patients’ immunonutritive status may improve the prediction of prognosis in HCC. One approach to combining immunonutritive scoring and liver synthesis as a nutrition parameter is the Prognostic Nutritive Index (PNI) [31]. The PNI comprises the total lymphocyte count and the albumin level and was first used in 1984 on patients undergoing gastrointestinal surgery [32]. Over recent decades, it has been identified as a prognosis prediction parameter for various cancer entities [31]. For patients with HCC, initial evaluation of the index also yielded promising results [33,34,35,36]. However, the role of the PNI in patients undergoing non-surgical treatment, especially in patients treated with locoregional therapy, has hardly been investigated [37,38,39]. Lately, we were able to prove the promising role for patients undergoing TACE [39].

Recently, Pan et al. were able to show that in particular, the combination of ALBI and PNI may be an appropriate stratification tool for patients with HCC undergoing ablation [40]. However, evaluations of this approach for other locoregional therapeutic options are lacking.

Therefore, this study aimed to investigate the role of a combination of ALBI and PNI for prognostication in patients with HCC undergoing TACE and to compare it to existing scoring systems.

## 2. Materials and Methods

This study was approved by the ethics committee of the Medical Association of Rhineland Palatinate, Mainz, Germany, for the retrospective analysis of clinical data (permit number 2021-15666). The requirement for informed consent was waived. Patient records and information were anonymized and de-identified prior to analysis. TRIPOD guidelines were followed for the writing process [41].

### 2.1. Patients

**Figure 1:**
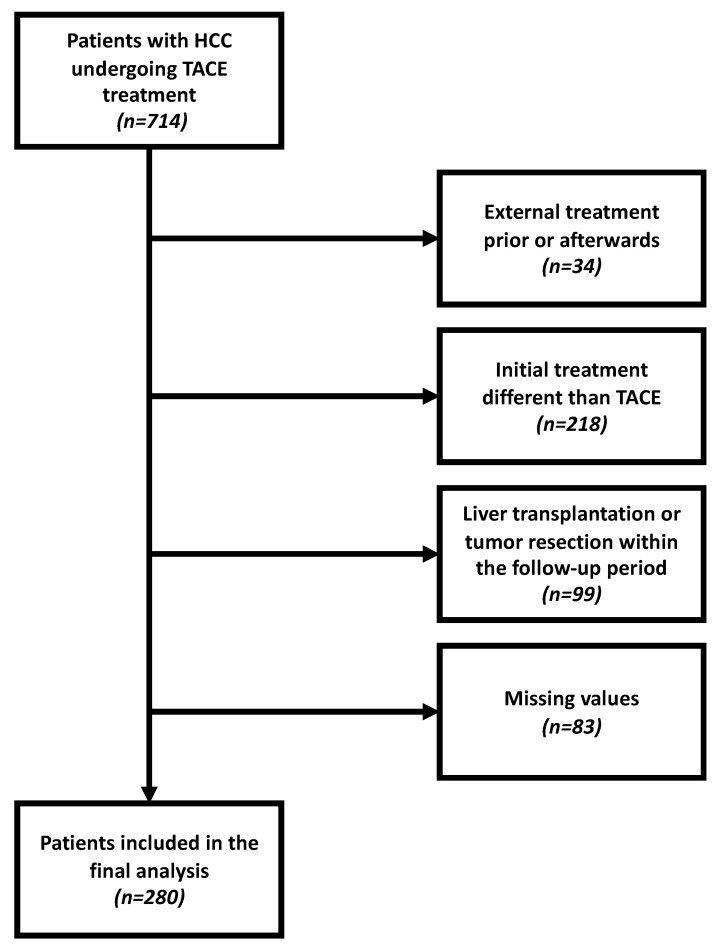
Flowchart of the inclusion of patients for whom the ALBI-PNI score could be evaluated.

Between January 2010 and November 2020, a total of 714 patients with confirmed HCC were referred to our tertiary care center for TACE treatment. For the various reasons provided in Figure 1, 434 of these patients had to be excluded. Consequently, 280 treatment-naïve patients with complete laboratory and imaging data were included in the final analysis. Follow-up ended on 31 March 2021.

### 2.2. Diagnosis, Treatment, and Follow-Up

HCC diagnosis was based on histological or image-derived criteria according to the EASL or AASLD guidelines [1,2]. All patients underwent contrast-enhanced CT or MRI prior to their first TACE treatment. As reported previously, patients were followed up with continuously repeated cross-sectional imaging within standardized time intervals [39]. Prior to the first treatment, all patients underwent an extensive discussion in an interdisciplinary tumor board consisting of hepatologists, oncologists, visceral surgeons, pathologists, radiation therapists, and diagnostic and interventional radiologists. The TACE itself was performed in a standardized manner as described elsewhere [42,43]. The patients underwent either conventional TACE (cTACE) or drug-eluting bead TACE (DEB-TACE). The decision was made after thorough discussion in our interdisciplinary tumor board taking into account tumor load, remnant liver function, ECOG status and concomitant diseases. The primary endpoint was OS, which was defined as the time interval between the initial TACE session and death or last follow-up.

### 2.3. Data Acquisition

As previously reported, the dataset for drafting this manuscript was acquired using the clinical registry unit (CRU) [39]. The CRU is an established prospectively populated database collecting all data on patients with liver cancer treated at our tertiary care referral center [44]. The dataset used for further analysis included demographic data, liver disease status and etiology, laboratory parameters, TACE-related parameters, and information on the tumor burden, including the number of lesions, the diameter of the greatest target lesion, and the tumor growth pattern. The dataset was completed using the radiology information system and the laboratory database.

### 2.4. Calculation of Scores

**Figure 2:**
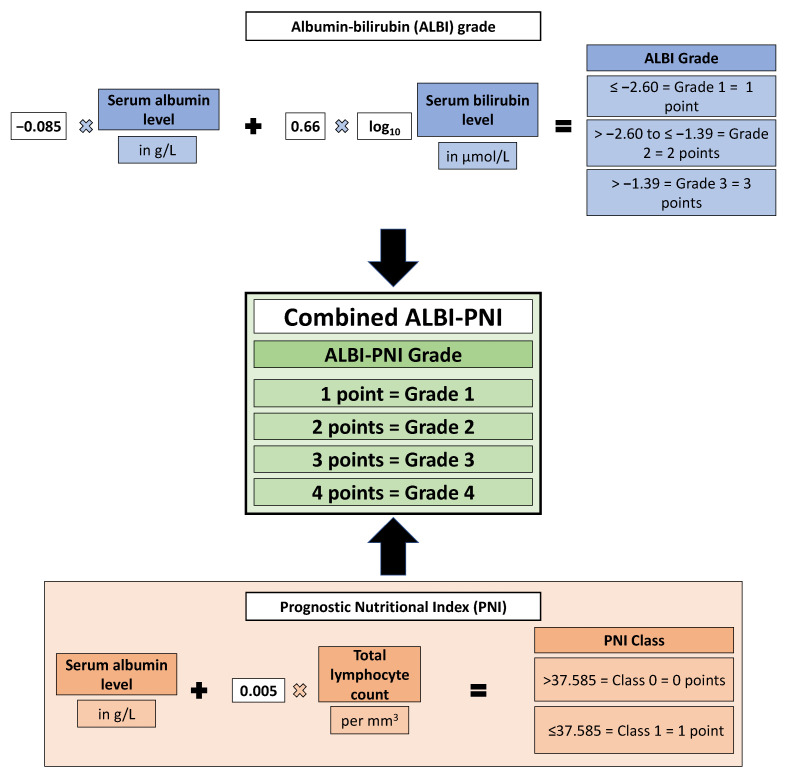
Overview of the formula for calculating the ALBI grade and PNI and the combination of both.

ALBI and PNI were calculated according to the original studies Figure 2 [25,33,34]. The PNI cut-off value was calculated using optimal stratification. The combined ALBI-PNI was calculated as follows: patients received a point value equal to their ALBI grade (1–3) and a value according to their PNI (high = 0 points; low = 1 point). These values were summed, resulting in an ALBI-PNI grade ranging from 1 to 4 points. The parameters included and their weighting factors are shown in Figure 2. BCLC, HAP, mHAP-II, and the Six-and-Twelve score were calculated as described in the original publications [3,8,16,17,18].

### 2.5. Statistical Analysis

Statistical analyses and graphics design were performed in R 4.0.3 (A Language and Environment for Statistical Computing, R Foundation for Statistical Computing, http://www.R-project.org; accessed on 31 January 2021).

Continuous data were reported as the median and range. Categorical and binary baseline parameters were reported in absolute numbers and percentages. Standardized cut-offs for the laboratory parameters were derived from our laboratory database. The packages “survminer” and “survival” (https://cran.r-project.org/package=survminer, Available online: https://CRAN.R-project.org/package=survival (accessed on 31 January 2021)). were used to carry out survival analyses, creating Kaplan–Meier curves and strata compared to the log-rank testing. Multivariate Cox proportional hazards regression models assessing hazard ratios (HRs) and corresponding 95% confidence intervals (CIs) were used to determine risk stratification and to evaluate the roles of the included factors. For further comparisons of the score with existing scoring and staging models, Harrell’s C concordance index (C-Index) was calculated using the “Hmisc” package (Available online: https://cran.r-project.org/package=Hmisc (accessed on 31 January 2021)). A C-Index of 0.5 indicates no predictive ability, and a C-Index of 1.0 indicates perfect predictive power [45]. Prediction error curves were based on the Brier score (package “pec”, Available online: https://cran.r-project.org/package=pec (accessed on 31 January 2021)). The Brier score at specific timepoints was defined as the mean squared difference between the observed outcome and the predicted outcome probability [46]. As a summary of the prediction error, the integrated Brier score (IBS) over the interval (0 months, 60 months) was calculated. A *p*-value < 0.05 was considered significant for all tests.

## 3. Results

### 3.1. Baseline Characteristics

The characteristics of the 280 patients analyzed are given in Table 1. Among the patients, 245 (87.5%) had liver cirrhosis with alcohol as the major etiology.

### 3.2. Survival Analysis of ALBI Grade and PNI

**Figure 3:**
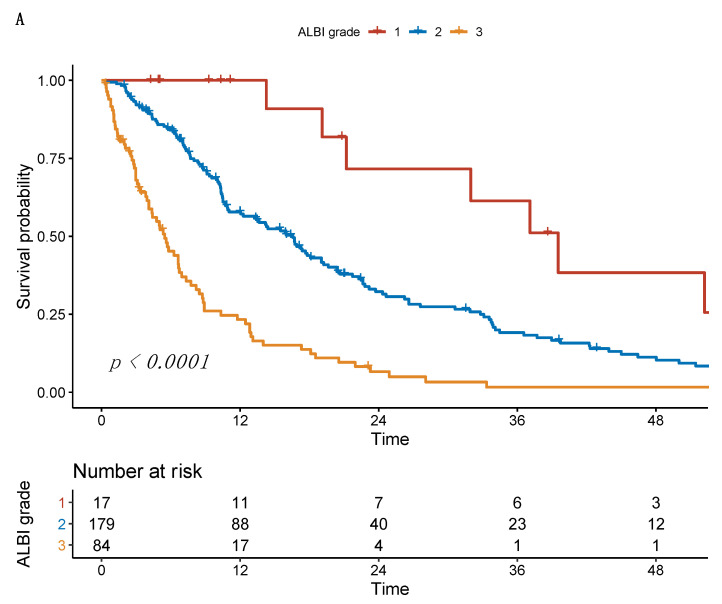

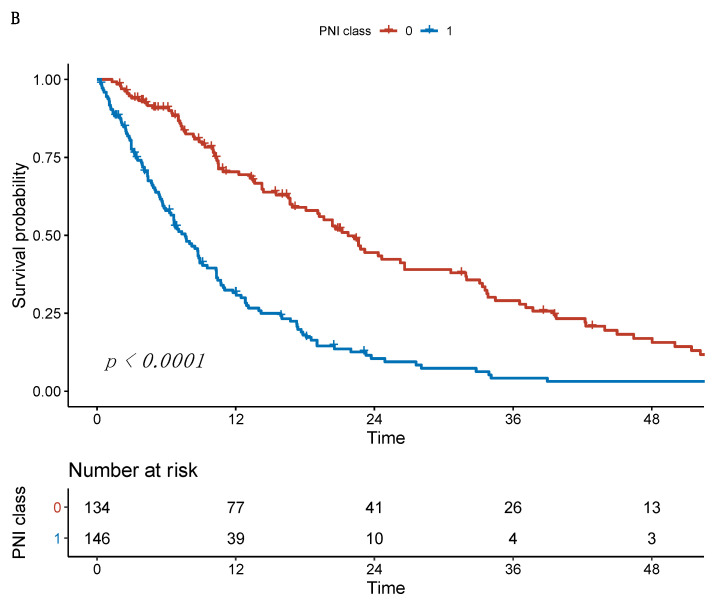
Kaplan–Meier curves of overall survival stratified according to (**A**) the ALBI grade and (**B**) the PNI.

Among the 280 patients, 17 (6.1%) had ALBI grade 1179 (63.9%) grade 2, and 84 (30.0%) grade 3. In the univariate analysis, the median OS for ALBI grades 1–3 was 39.0 months, 16.3 months, and 5.4 months, respectively (*p* < 0.001). The optimal cut-off value for the PNI was 37.59 points using optimal stratification. According to this value, 134 (47.9%) of the patients had a low PNI and 146 (52.1%) had a high PNI. Patients with a low PNI had significantly impaired median OS compared to patients with a high PNI (7.5 months vs. 21.4 months, *p* < 0.001), see Figure 3.

Univariate Cox hazard regression models revealed significant *p*–values for ALBI grade 3, low PNI, high aspartate aminotransferase (AST) level, and multifocality. None of the other included risk factors reached significance. In the multivariate analyses including all of the significant factors, only a higher ALBI grade and low PNI remained independent prognostic factors (Table 2).

### 3.3. Survival Analysis Using ALBI-PNI Grade

**Figure 4:**
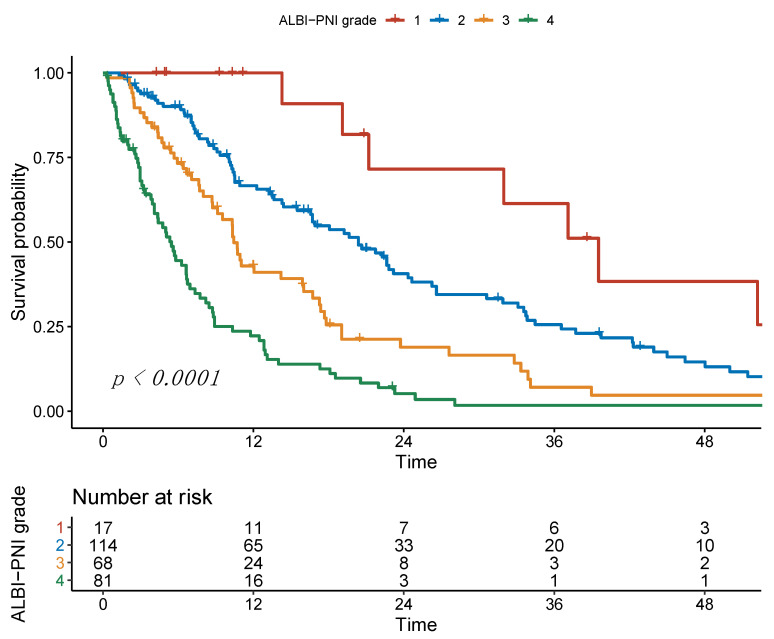
Kaplan–Meier curves of overall survival stratified according to the ALBI-PNI grade.

After combining ALBI and PNI, 17 (6.1%) patients had an ALBI-PNI grade of 1, grade 2 in 114 (40.7%), grade 3 in 68 (24.3%) and grade 4 in 81 (28.9%) of the patients. The median OS was 39.0 months, 20.1 months, 10.3 months, and 5.4 months, respectively (*p* < 0.001). The *p*-values in pairwise comparisons were 0.023, 0.001, and <0.001 for grade 1 vs. grade 2, grade 2 vs. grade 3, and grade 3 vs. grade 4 (Figure 4). Regarding the C-Indices, the combined ALBI-PNI (C-Index 0.69) was superior to ALBI (C-Index 0.65) and PNI (C-Index 0.64) alone (Table 3).

**Figure 5:**
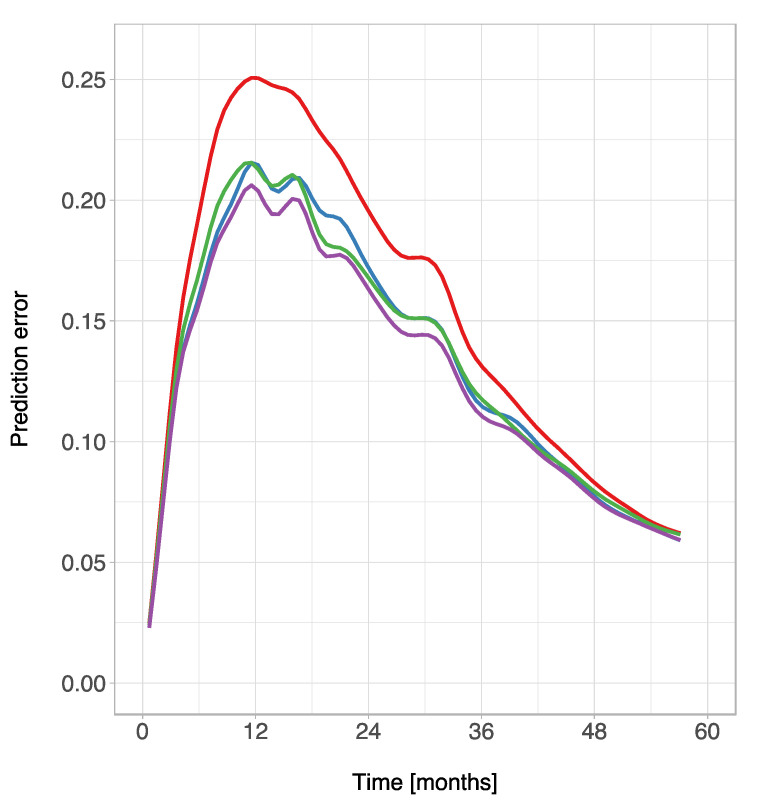
Prediction error curves for Kaplan–Meier estimates based on the ALBI grade (blue), PNI (green), ALBI-PNI grade (purple), and the unstratified sample (red).

The IBS over the interval (0 months, 60 months) was 0.131 for ALBI, 0.132 for PNI, and 0.126 for ALBI-PNI. In comparison, the IBS was 0.150 using the Kaplan–Meier estimates for the unstratified sample. Prediction error curves based on the Brier score are shown in Figure 5.

### 3.4. Comparison of the ALBI-PNI to Existing Scoring Systems

Compared to the established scoring and staging systems BCLC, HAP, and mHAP-II, the ALBI-PNI was superior in survival prediction. Table 4 provides a detailed overview of the comparison.

## 4. Discussion

In this study, the ALBI grade and PNI were both strong independent predictors of survival. However, the combination of the two scores allowed for even more precise predictions. Furthermore, the ALBI-PNI had the best predictive performance for our cohort in direct comparisons to established prediction systems.

Several studies have identified the ALBI grade as an independent prognostic factor for median OS in patients with HCC undergoing TACE, and as a more precise stratification system than the CPS [25,26,27,47,48,49,50]. However, in a recent head-to-head comparison with several established scoring systems, it missed significance [12]. Thus, the stratification ability of the ALBI grade may be improved by combining it with other risk-associated factors. One recently investigated option is the P-ALBI, combining albumin, bilirubin, and the platelet count. Although Ni et al. concluded that the P-ALBI outperforms the ALBI grade alone, the results from Carling et al. do not confirm this [27,28]. In our study, a low platelet count was associated with impaired survival (HR = 1.3, 95% CI 0.9–1.7), but without reaching statistical significance in univariate analysis (*p* = 0.140). Therefore, platelet count is outperformed by other risk factors and would not have added any value to the ALBI grade’s performance.

A novel combination partner for ALBI was recently proposed by Pan et al.: They developed the immunonutritive scoring system PNI as a complementary factor and evaluated the new system for patients with HCC undergoing tumor ablation [40]. The PNI is an immunonutritive scoring system and incorporates albumin as a surrogate of the nutritive status and the total lymphocyte count as a surrogate for the patient’s immune status. For patients with an underlying liver disease albumin is even bifunctional and does not only cover the patient’s nutritive status: As albumin is an important indicator of the remnant liver function this even enhances the role of this parameter for the PNI and its predictive ability [23,24]. Lymphocytes function as an indicator of the counter-regulations of the immune system and play an essential role in tumor defense by inhibiting cell proliferation and migration. Inflammation in general has been identified as one of the key drivers of cancer development and progression [29,51]. For HCC, the inflammatory process plays an even more important role, as the chronic inflammation and tissue remodeling create an ideal microenvironment for cell transformation and pathways leading to unregulated proliferation [51]. Consequently, the PNI has been identified as a profound prognostic factor and good stratification tool for patients with HCC undergoing surgery or systemic treatment [33,34,35,36].

However, literature on the PNI in patients undergoing TACE is scarce, as only three studies are available on this issue. First, Liu et al. identified the PNI as a strong predictor of OS in univariate analysis, but their analysis lacks statistical power because, despite being a significant factor for impaired OS, they did not include the factor in the multivariate analysis [37]. Second, He et al. identified the PNI as a highly predictive factor for median OS in a univariate analysis for patients treated with a combination of TACE and recombinant human type-5 adenovirus H101 [38]. Even though patients with a higher index had superior survival (HR = 0.685), the PNI did not reach significance in the multivariate analysis (*p* = 0.091). In a recent study, we identified the PNI as the favorable immunonutritive scoring system for Western patients undergoing TACE [39]. While most studies on immunonutritive scoring have been conducted on Asian patients with viral hepatitis being the major etiology, in the present study, alcoholic liver disease was the main trigger. Therefore, our results prove the role of the PNI as an important stratification tool regardless of the underlying etiology or the ethnical background.

The combination of ALBI and PNI seems to be not only additive, but complementary, as Pan et al. recently demonstrated superiority of the ALBI-PNI for patients with early stage HCC compared to ALBI and PNI alone [40]. In our study, we confirmed the promising results for patients undergoing TACE. Furthermore, our study is the first to compare the combination of ALBI and PNI to existing scoring systems. As the ALBI-PNI was the best performing model for our cohort, the system may also have value as an additional easy-to-calculate meta-parameter of hepatic function, nutritive status, and immune response in daily clinical routine.

The big advantage of the ALBI-PNI is that all included parameters are easily accessible and part of the standard evaluation during the workup for TACE. Compared to other diagnostic tools, the included laboratory parameters are cheap and do not generate an additional logistic effort. Thus, implementation of the ALBI-PNI in daily clinical routine is extremely simple. Due to the objective and quantitative character of laboratory parameters, the ALBI-PNI does not include any subjective estimations. Thus, the score has an additional advantage of high reproducibility.

Despite a higher ALBI score and a low PNI, a high AST level and multifocality were prognostic factors in univariate analysis. However, none of them kept significance in multivariate analysis. Thus, the prognostic power of the Six-and-Twelve score as a score only based on tumor burden was lower than that of the other established scoring systems. This is in line with previously reported external validation results of the Six-and-Twelve score, which indicate that especially scoring systems incorporating both tumor burden and liver function perform the best [13]. Therefore, future studies should evaluate a combination of the ALBI-PNI with indicators of the tumor burden.

This study has several limitations. First and foremost, it was carried out as a single center study with only a moderate sample size (*n* = 280). This may be due to our decision to only include patients for whom all values were available and to not impute missing values in order to maintain high data quality. In addition, we only included patients from 2010 onwards to ensure comparability in the diagnostic and follow-up workflow of the patients and to guarantee standardization in the TACE procedure itself. Nevertheless, the study size was still comparable to other existing studies on the issue. Secondly, we decided to exclude patients who underwent curative therapies after TACE because we aimed to ensure a higher degree of homogeneity within the cohort in order to avoid bias [12]. Thirdly, the study was performed not only on ideal TACE candidates in BCLC stages A and B; we actively decided to include patients with more advanced stages because it would represent a real-world situation and take into account the widely accepted concept of stage migration [1]. Fourthly, we did not perform any subgroup analysis of patients treated with different TACE techniques. However, multiple comparisons between cTACE and DEB-TACE have not shown any influence on OS [52,53,54]. Moreover, recent observations have proven that the predictive power of scoring systems is equivalent in cTACE and DEB-TACE [55]. Finally, universally accepted cut-offs for the PNI are not available. Therefore, we used optimal stratification methods to identify optimal cut-offs. However, the cut-off values used in our study fell within the range of values used on other studies conducted for Western patients [38,56,57,58]. Nevertheless, future multicenter studies are mandatory to define universal cut-off values for Caucasian patients.

## 5. Conclusions

Both scoring systems, ALBI and the PNI, were highly predictive prognostic factors. However, the combination of both was superior in survival prediction. Furthermore, ALBI-PNI outperformed all of the established scoring and staging systems. Thus, ALBI-PNI may be a new stratification tool for patients with HCC undergoing TACE, reflecting both immunonutritive status and liver function. Due to its objectivity and easy calculation, ALBI-PNI may support decision-making in such patients. However, future validation of the grading is needed prior to implementation in a daily clinical routine.

## Figures and Tables

**Table 1:** Baseline characteristics of patients with HCC undergoing TACE included in this study.

Variable	All Patients (*n* = 280)
Age, years	69.5 (62.5–75.4)
Gender	
Female	46 (16.4%)
Male	234 (83.6%)
Etiology, *n*	
Alcohol	131
Hepatitis C	46
Hepatitis B	26
NASH	27
Hemochromatosis	5
AIH/PBC/PSC	5
Unknown/Other	40
Child-Pugh Stage	
A	104 (37.2%)
B	116 (41.4%)
C	25 (8.9%)
No cirrhosis	35 (12.5%)
BCLC Stage	
0	0 (0%)
A	45 (16.1%)
B	154 (55.0%)
C	58 (20.7%)
D	23 (8.2%)
Max. tumor size, cm	4.2 (2.9–6.4)
Tumor Number	
Unifocal	55 (19.6%)
Multifocal	203 (72.5%)
Diffuse growth pattern	22 (7.9%)
Albumin level, g/L	31 (27–35)
Lymphocyte count, per mm^3^	1214 (834–1558)
Bilirubin level, mg/dL	1.3 (0.8–2.2)
Platelet count, per nL	128 (87–193)
AST level, U/L	64.5 (47.0–95.5)
ALT level, U/L	41.5 (28.0–61)
INR	1.2 (1.1–1.3)
AFP level, ng/mL	45 (8.1–777.0)
Type of TACE	
cTACE	95 (33.9%)
DEB-TACE	185 (66.1%)

Values are given as *n* (%) or median (interquartile range) unless otherwise noted. NASH, nonalcoholic steatohepatitis. AIH, autoimmune hepatitis. PBC, primary biliary cholangitis. PSC, primary sclerosing cholangitis BCLC, Barcelona Clinic Liver Cancer. AST, aspartate aminotransferase. ALT, alanine aminotransferase. AFP, alpha fetoprotein. cTACE, conventional transarterial chemoembolization. DEB-TACE, drug-eluting bead transarterial chemoembolization.

**Table 2:** Univariate and multivariate Cox proportional hazards regression models evaluating PNI, ALBI grade, and other risk factors.

Analysis		Univariate			Multivariate		
Covariate		HR	95% CI	*p*-Value	HR	95% CI	*p*-Value
ALBI grade	1	Reference			Reference		
	2	2.6	1.3–5.3	0.010	2.2	1.0–4.7	0.053
	3	7.3	3.5–15.3	<0.001	3.8	1.6–8.9	<0.001
PNI class	0	Reference			Reference		
	1	2.7	2.0–3.5	<0.001	2.0	1.4–2.9	<0.001
Age	≥70 years	1.0	0.8–1.3	0.960			
AFP	>400 ng/mL	0.9	0.7–1.2	0.620			
AST level	>31 U/L	2.0	1.1–3.7	0.025	1.6	0.9–3.0	0.136
ALT level	≥35 U/L	1.2	0.9–1.6	0.200			
INR level	>1.2	1.1	0.8–1.5	0.460			
Platelet count	<150/nL	1.3	0.9–1.7	0.140			
Tumor number	≥2	1.5	1.0–2.1	0.027	1.2	0.8–1.7	0.423
Max. lesion size	>5.0 cm	1.3	1.0–1.7	0.058			

AFP, alpha fetoprotein. AST, aspartate aminotransferase. ALT, alanine aminotransferase.

**Table 3:** Head-to-head comparison of the ALBI grade alone, the PNI alone, and the combination of both.

Score		Median OS	HR	95% CI	*p*-Value	C-Index
ALBI	1	39.0	Reference			0.65
	2	16.3	2.6	1.5–5.3	0.010	-
	3	5.4	3.2	3.5–15.3	<0.001	-
PNI	0	21.4	Reference			0.64
	1	7.5	2.7	2.0–3.5	<0.001	-
ALBI-PNI	1	39.0	Reference			0.69
	2	20.1	2.2	1.0–4.5	0.037	-
	3	10.3	3.8	1.8–8.0	<0.001	-
	4	5.4	7.8	3.7–16.4	<0.001	-

OS, overall survival; HR, hazard ratio; CI, confidence interval.

**Table 4:** Head-to-head comparison of the ALBI-PNI and different scoring systems.

Score		Median OS	HR	95% CI	*p*-Value	C-Index
ALBI-PNI	1	39.0	Reference			0.69
	2	20.1	2.2	1.0–4.5	0.037	-
	3	10.3	3.8	1.8–8.0	<0.001	-
	4	5.4	7.8	3.7–16.4	<0.001	-
BCLC	A	31.5	Reference			0.66
	B	14.1	1.7	1.1–2.6	0.011	-
	C	6.4	3.2	2.1–5.1	<0.001	-
	D	4.9	4.5	2.5–8.1	<0.001	-
HAP	A	39.0	Reference			0.60
	B	15.7	2.1	1.1–4.1	0.031	-
	C	11.9	2.5	1.4–4.6	0.003	-
	D	8.7	3.1	1.7–5.8	<0.001	-
mHAP-II	A	41.7	Reference			0.59
	B	15.7	2.3	0.9–5.8	0.070	-
	C	14.2	2.3	1.0–5.4	0.051	-
	D	10.2	3.6	1.6–8.2	0.002	-
Six-and-Twelve	1	14.1	Reference			0.55
	2	13.8	1.1	0.8–1.5	0.650	-
	3	7.6	2.2	1.5–3.3	<0.001	-

OS, overall survival; HR, hazard ratio; CI, confidence interval.

## Data Availability

Data cannot be shared publicly because of institutional and national data policy restrictions imposed by the Ethics Committee of the Medical Association of Rhineland Palatinate, Mainz, Germany, since the data contain potentially identifying patient information. Data are available upon request for researchers who meet the criteria for access to confidential data.
